# Beware the “loneliness gap”? Examining emerging inequalities and long‐term risks of loneliness and isolation emerging from COVID‐19

**DOI:** 10.1002/ajs4.223

**Published:** 2022-06-23

**Authors:** Roger Patulny, Marlee Bower

**Affiliations:** ^1^ Arts Social Science and Humanities University of Wollongong Wollongong New South Wales Australia; ^2^ The Matilda Centre for Research in Mental Health and Substance Use, Faculty of Medicine and Health The University of Sydney Sydney New South Wales Australia

**Keywords:** covid‐19, emotional loneliness, friendship, loneliness, social capital

## Abstract

Emerging evidence suggests COVID lockdowns have not only increased the social problem of loneliness but widened the ‘loneliness gap’ between the most and least lonely people. Qualitative investigation can reveal why this gap might have increased, for whom, and whether the loneliness gap will remain long term. Using multi‐wave qualitative survey data conducted during Australia’s 2020 lockdown period and beyond, we examine personal experiences of interaction transitioning out of lockdown. We find substantial and uneven impacts of COVID lasting well beyond lockdown. Participants reported heightened loneliness attributable to: physical isolation, health anxieties, ceased activities, reduced connection quality, and poor motivation. COVID also created new interactive difficulties for singles, those with physical and mental disabilities, their carers, and those with low social capital. There was also reported ‘pruning’ of social networks (i.e. reduced bridging, increased bonding social capital), and evidence that increased digital interaction did not substitute for lost physical contact. Younger people also experienced isolating COVID‐induced life disruptions (e.g. travel, university attendance etc). Findings suggest COVID has increased potential long‐term inequalities in loneliness, highlight the post COVID risks faced by vulnerable groups, and suggest caution in advocating digital solutions as a panacea for diminished physical interaction in the post‐pandemic world.

## 1 INTRODUCTION

The longer‐term effects and inequities of COVID‐19 on social interaction and loneliness as the pandemic recedes are unclear. While the virus clearly had an enormous impact on social interaction during lockdowns globally, a critical question remains as to its long‐term impact: did changes in social interaction during lockdown impact some more severely than others, and have they instigated a long‐term decline in social interaction and increase in loneliness? The potential consequences are troubling: loneliness is associated with poor health outcomes including early mortality (Holt‐Lundstad et al., 2015), mental health and depression (Nangle et al., 2003), suicide (Kidd, 2004) and high social and healthcare costs (Mihalopoulos et al., 2019). It can be defined as a discrepancy between a desired and available relationships, or a lack of *quality* relationships that provide meaningful interaction and support (Weiss, 1973).

It has been observed in several studies that COVID‐19 increased loneliness in the general population through the isolating effects of lockdown and restrictions on activities (Kok et al., 2022; Stickley & Ueda, 2022; Zaninotto et al., 2022). A recent UCL‐UK study confirms this (WWCfW, 2020), and also charts disparities in *who* experienced lockdown loneliness. The least lonely people became temporarily less lonely while the loneliest people became *even lonelier* during lockdown. This suggests COVID‐19 lockdown may have generated new inequalities and even an increased “loneliness gap.”

However, while the study hints at loneliness worsening for certain groups – e.g. university students – it does not delve into participant experiences and reasons to help us understand *why* this gap might have worsened for certain groups and not others. In a wide‐ranging pre‐COVID assessment, De Jong Gierveld et al. (2016) identify several groups with a higher prevalence of loneliness, including the young and old, women, those with lower education and income, poorer health, migrant status, separated and/or lacking proximate kin. How has COVID impacted such groups of people and their experiences? Were these experiences changing even before COVID struck? Or have existing studies been able to establish if there are longer‐term factors impacting social interaction that might lead to more long‐term changes in loneliness than that the UCL‐UK quantitative data immediately suggest. There is potential that changes endemic to COVID may have entrenched longer‐term norms of reduced physical contact, and a potential “culture of loneliness” (Patulny & McKenzie, 2021).

Any claims of COVID‐induced impacts on vulnerable groups, or contributions toward rising cultures of loneliness, must account for ongoing and uneven social changes that were underway prior to COVID‐19. COVID may represent an *acceleration* of a prior uneven trend toward reduced physical interaction and greater loneliness, as late modern societies became more individualised and liquid (Archer, 2012; Bauman, 2005; Giddens, 1990), and as civil society and volunteering declined (Putnam, 2000).

However, such changes must be offset against the digital disruption that preceded COVID, and then accelerated dramatically in the last 2 years with the stellar rise in digital communication technology and videoconferencing. Such changes are here to stay, and their impact is unclear. When digital interaction “displaces” physical connections, loneliness typically worsens (Nowland et al., 2018). The combined longer‐term effect of decreasing physical interaction and increasing digital interaction is therefore uncertain, and may lead to longer‐term changes in cultures of interaction (Patulny & McKenzie, 2021). If digitisation negatively impacts those already vulnerable to isolation, the long‐term “loneliness gap” may widen further still.

To explore these questions, we need to ask people about their experiences during and after lockdown. This study looks at how interaction, lifestyles and trajectories changed during COVID‐19, who was impacted and if this is indicative of lasting changes and inequities in how we socialise and interact.

## 2 LATE MODERN LONELINESS – ACCELERATED AND UNEQUAL LONELINESS?

### 2.1 Individualised lifestyles in late modernity

Sociological theories of late/liquid modernity from Giddens (1990) and Bauman (2005) discuss the idea of a long‐term change in the way people interrelate and lead their lives. Whether depicted more positively as an emancipatory liberating movement (Giddens, 1990) or more negatively as precursor to general social malaise (Bauman, 2005), these theories highlight the rise in more individualised lifestyles emphasising choice, consumption, transformation, freedom and self‐responsibility (Archer, 2012; Davis, 2008). However, this “individualisation” may lead us to forego the advantages of social bonds as well as the constraints. Individualisation has been linked to an increased risk of loneliness as traditional supportive social bonds are weakened (Franklin et al., 2019; Hookway et al., 2019).

Depictions of late modern loneliness are complicated by the existence of multiple forms of loneliness. Weiss (1973) differentiated between *social loneliness,* or the absence of a satisfying larger social group who provide often diffuse forms of support, such as friendships or activities, and *emotional loneliness*, or the absence or loss of close attachment relationship/s who provide strong emotional support such as a partner or immediate family (DiTommaso & Spinner, 1997; Hood et al., 2018). There is also *collective loneliness*, or the sense of isolation experienced when people become disconnected and alienated from an “outermost social layer” of their network (Dunbar, 2014), such as a broad community, people, identity group or nation (Caccioppo et al., 2015). Lockdown (and its aftermath) may impact these lonely experiences differently, depending on the kinds of vulnerabilities we take into lockdown (e.g. lacking in digital skills or social capital) and the kind of people lockdown restricts us from interacting with (e.g. partners, parents, colleagues and community groups).

Late modern loneliness is complicated not just from a loss of tradition, but from parallel cultural and structural transformations, including increased family/relationship diversity and single people; changing work patterns and collegial relationships; and declining civic activity and social capital. We briefly review these structural factors in terms of how they relate to vulnerability to loneliness in late modernity, and the likely impact COVID‐19 and its aftermath might be having on this relationship.

### 2.2 Late modern structural transformations impacting loneliness before and during COVID‐19

An important characteristic of late modernity is a change in family forms and relationships away from the “heterosexual nuclear family” that dominated the mid‐20th Century Western cultures, toward more diverse sexualities and relationship forms (Giddens, 1990). This includes shrinking families, increasing single‐person households and greater family mobility in pursuit of economic/work opportunities (Therborn, 2014). Such changes disrupt known protective factors of loneliness, including strong family ties (Das, 2021), having an intimate partner (De Jong Gierveld et al., 2016) and relationship stability (particularly among younger people) (Hookway et al., 2019), and suggest a potential loneliness gap between single and couple households. Lockdowns would increase emotional loneliness, at least temporarily, by restricting the capacity to visit kin and friend relations who live separately, with disproportionate impact on single persons. This is reflected in evidence of increased loneliness during COVID among (largely single) young people (Lee et al., 2020), and people living alone with mental illness (Heron et al., 2022), and new parents in COVID who reported heightened depression from a loss of important support networks (Myers & Emmott, 2021). There are mixed findings concerning a widening loneliness gap around issues of family and ageing. Some studies find an initial age‐related loneliness gap with younger people typically doing worse during COVID (Stickley & Ueda, 2022), although other studies find no evidence that this gap increased during COVID (Moreno‐Agostino et al., 2022). Other studies found a widening gap in loneliness during COVID based on gender (i.e. worse for women), and for people living alone in particular (Zaninotto et al., 2022).

Similarly, changing work and education patterns have impacted social relations. Late modern workers are increasingly required to be flexible in work arrangements and hours, and willing to relocate in pursuit of work in an increasingly contract‐based, globalised capitalist economy (Salazar & Shiller, 2014; Urry, 2000). Social disconnection has been observed in the physical and intergenerational “drift” between colleagues in modern flexible workplaces with high staff turnover (Sennett, 1998) and the disempowerment of modern globalised precariats (Standing, 2013). COVID‐19 has dramatically transformed work arrangements, substantially increasing the practice of working from home via digital means (Nagel, 2020) and expanding digital aspects of the gig economy (Spurk & Straub 2021). This may increase the loneliness gap between digital and non‐digital workers, with reports already emerging of worker alienation in the emerging platform economy (Subramony et al., 2018). Protracted unemployment and churn produced by COVID may also prise open a loneliness gap between the employed and unemployed: prior research finds long‐term unemployed people need to manage high degrees of loneliness and isolation (Peterie et al., 2019), while other research finds heightened loneliness among socioeconomically disadvantaged persons, both in general (Stickley & Ueda, 2022) and among those with a mental illness (Heron et al., 2022). Zaninotto et al., (2022) find greater loneliness among those with less wealth both prior to and during COVID, but that the loneliness gap between these two narrowed during COVID.

Late modernity is also characterised by reduced socialising, civic activity and volunteering in general. Putnam (2000) and contemporaries (e.g. Saracino & Mikucka, 2017) propose that voluntary activity and social capital are declining across western societies, shifting away from interacting in community groups toward more isolated, often solo activities.

Such changes and their impact on loneliness are complicated by the presence of different forms of social capital; i.e. open civic bridging networks, and exclusive, private bonding networks (Patulny & Svendsen, 2007; Putnam, 2000). While bonding and bridging are perhaps more heuristic than definitive as concepts, each implies a useful relationship between tie strength and access to resources – such as information (bridging) and support (bonding) – for understanding loneliness during COVID. This relationship is not universal; competing conceptions of social capital including Bourdieu's (1986) exclusive class‐based conception; vertical and horizontal ties (Ryan, 2011); and cultural variants such as Chinese guanxi (Feng & Patulny, 2021) offer alternative dynamics around ties, solidarity, preexisting resources and resource sharing. However, previous studies have identified bonding and bridging social capital constellations of greater and weaker tie strength in Australia (Patulny, 2015; Stone & Hughes, 2002), while other studies have linked tie strength to loneliness (Dunbar, 2014).

Taking such studies together in the COVID context of disrupted networks, we can speculate that a loss in bonding social capital might increase emotional and social loneliness because of its personalised nature, while a loss in bridging social capital could increase social and collective loneliness by eroding the capacity to connect to new people and communities (including strong identity‐based connections). The uneven distribution of either forms of capital among populations and between individuals is also a likely contributor to the loneliness gap.

## 3 DIGITAL INTERACTION IN LATE MODERNITY, COVID‐19 AND BEYOND

One of the most important transformations in late modern society is a rapid increase in digital interaction (Patulny & Olson, 2019), which has been greatly accelerated by COVID‐19. Pre‐COVID‐19 research revealed increasing digital connectivity is matched by declining face‐to‐face connections (Patulny & Seaman, 2017), with yet unknown consequences for societal loneliness. The literature around the impact of digital technology is mixed: some posit a *displacement* effect, where often poorer‐quality digital interaction replaces more authentic and supportive physical relations, versus a *stimulation* effect where digital engagement supports existing relations and encourages new connections (Nowland et al., 2018).

These countervailing effects can depend on preexisting social resources and practices, including community engagement, support (Kraut et al., 2002), attachment (Benoit & DiTommaso, 2020), online engagement style (passive versus interactive) (Yang, 2016) and instrumental exchange versus community‐oriented relationships (Matook et al., 2015). An uneven distribution of such resources and practices may further widen the loneliness gap.

### 3.1 Digital impacts on family, work and social capital during and after COVID‐19

COVID‐19‐induced increase in videoconferencing, social media and online gaming may help some people compensate for the loss in face‐to‐face interaction during lockdown and beyond, and “stimulate” the development of new connections to reduce social and collective loneliness. However, they may also increase loneliness for others if they “displace” important physical world connections.

COVID‐19‐induced increases in family household time (through enforced lockdown) and videoconferencing of distant relatives have likely helped with the emotional loneliness of older people; Sum et al. (2008) link increased higher internet use among older people to reduce “familial” emotional and social loneliness when used to communicate with existing friends and family. However, the same dynamics are unlikely to apply for younger people already saturated in this form of interaction (Teppers et al., 2014), which might result in an age gap in loneliness.

Increased digital teleworking pits a loss of in‐person socialising with colleagues and customers against an increase in digital collegial interactions. Digital work may have an additional long‐term positive impact on social and emotional loneliness by reversing the need for work‐related mobility and commuting, and thus the “drift” from families and communities of origin (Sennett, 1998). Alternatively, workers may form fewer friendships through colleagues, or around important work issues related to their identity, potentially increasing social and collective loneliness. Precarious and long‐term unemployed workers with little choice over digital or physical work options will have reduced options in navigating between these worlds and choices, potentially contributing to an employment‐based loneliness gap.

Existing research shows mixed associations among digital interaction, social capital and loneliness, making projections of post‐COVID‐19 social networks difficult. Bridging social capital can facilitate online interaction, allowing Facebook users (for example) to access new people, ideas and worldviews (Lampe et al., 2013), although expansive networks are unlikely to stimulate connections and reduce loneliness if they are passive (lurking) rather than active (interactive) (Burke et al., 2010). Either way, those lacking preexisting social capital connections are likely to become lonelier after lockdown.

### 3.2 Changes and inequalities in loneliness beyond COVID‐19

It is unclear whether COVID‐induced changes in family, work and social networks have had a long‐term impact on loneliness beyond the impacts of late modernity, or whether these changes have impacted some groups more strongly than others and opened up loneliness gaps. This is the topic of our present investigation. It leads us to the following exploratory research question which will investigate through analysis of a unique qualitative dataset of Australians during and after COVID‐19 lockdown:

RQ – “How have the physical restrictions and increased digital interactions associated with COVID‐19 affected inequalities in loneliness and long‐term social and community connections, relationships, and activities?”

## 4 DATA AND METHOD: QUALITATIVE SURVEY ANALYSIS

### 4.1 Data

People’s experiences of the first Australian lockdown, which began on 23 March 2020, began to ease across the country in late April and early May 2020 (depending on the state of residence). By mid‐2020, most of the Australian population emerged from lockdown into a “post‐COVID‐19” future characterised by minimum restrictions and lockdowns, lasting approximately 12 months (Australian Department of Health, 2021). This early exit (relative to other countries) afforded us a unique opportunity to explore participant experiences over an extended period beyond lockdown.^1^ One exception to this was residents of Victoria for whom a subsequent COVID outbreak led to a reinstatement of lockdown from 8 July 2020 to 27 October 2020.

Qualitative data were collected as part of a baseline survey of a longitudinal study exploring the prevalence and impacts of COVID‐19‐related social and economic changes on mental health among Australian adults (approved by XXXX Ethics Committee). Data were extracted from open‐ended responses to the following questions: “What impact has the COVID‐19 pandemic had on your mental health, emotions and/or wellbeing?”; “What impact, if any, has the COVID‐19 pandemic had on your social relationships and/or the way you socialise?”; “What impact has the COVID‐19 pandemic had on the way you feel about your housing situation and the surrounding neighbourhood?” and “Please comment on any other experiences or changes you have had as the result of the COVID‐19 pandemic.”

The number of days since lockdown was calculated for each participant based on the date that they completed the survey and the number of days since residents in their state were released from lockdown; defined as when at least some recreational indoor and outdoor activities were permitted. The median number of days since lockdown for included participants was 73 (r: 231, SD: 65.4). It is worth noting that many Victorian participants classified as “in lockdown” at the time of survey were undergoing their second COVID‐19 lockdown period. Consequently, the experiences of this group may reflect the cumulative effect of iterative lockdowns, rather than the effect of a single lockdown.

The survey also included a quantitative question on loneliness, asking whether participants “felt lonely at least some of the time, or for a minimum of 1‐2 days per week.” This question will be examined briefly as a useful foreground for the qualitative results which are the main focus of this study.

### 4.2 Recruitment and participants

Participants were recruited between 7 July and 31 December 2020 primarily through self‐enrolment in the online survey through social media advertisements (e.g. Facebook or Instagram).^2^ Surveys took 30–60 minutes to complete. All participants were volunteers and could enter an optional $250 prize draw (voucher). The final dataset contained 6500 qualitative responses from 2065 participants. A subset of the total qualitative dataset was derived from phrases concerned with digital interaction, including “digit*”, “virtu*”, “app*” “online”, “internet”, “web”, “chat”, “text”, “Facebook”, “email”, “Zoom”, “FaceTime”, “real life”, “in‐person” and “face‐to‐face”, resulting in a subset of 1069 responses from 795 participants. Table 1 presents demographic characteristics of participants whose quotes were included in analysis (subset) and the total baseline sample. Subset and total sample characteristics were similar, except for gender where the subset had 10 per cent more females.

**TABLE 1:** Demographic characteristics of analysis subsample and total baseline sample

	Subset (*n* = 795)	Total (*N* = 2065)
Gender (female)	75.9%	66.3%
Age (median)	38 (r:18–88)	39 (r: 18–88)
Born in Australia	76.0%	75.2%
Residence (State/Territory)		
New South Wales	44.8%	44.5%
Victoria	33.7%	30.9%
Queensland	8.1%	9.2%
South Australia	4.2%	4.6%
Western Australia	3.0%	4.2%
Australian Capital Territory	4.3%	3.9%
Tasmania	1.8%	2.3%
Northern Territory	0.3%	0.5%
Lesbian, Gay, Bisexual, Transgender, Intersex, Queer or Asexual +	18.9%	17.1%
Employed (full‐time, part‐time or casual)	60.9%	56.2%

### 4.3 Analysis

NVivo 12 (QSR International, 2018) was used to store and code data. Thematic analysis (Braun and Clarke, 2006) was used for several reasons. First, its flexible nature (not extrinsically wedded to a particular theory) means it is more adaptable to answer our exploratory research question and facilitates linking findings with existing sociological theory, compared to Grounded Theory, which explicitly aims to develop new theory (Charmaz & Smith, 2003). Second, the form of our data – short qualitative responses – is better suited to identification of broader themes than closer reading of participant semantics or narratives, as in Discourse (Johnstone, 2017) or Narrative Analysis (Herman & Vervaeck, 2019).

Together, authors read through a quarter of the data, inductively coding data which reflected recurring thematic patterns. Codes were then organised loosely into draft themes. Through repeated readings, the themes shifted from descriptive (e.g. “negative examples of digital interaction”) to interpretative (e.g. “COVID‐19 relationships as shrunken and more intensified”). Authors then read the remaining dataset separately and continued refining the coding framework. They then collated their proposed amendments to the coding framework using an iterative process of reading, reflection and discussion. The entire dataset was then recoded to match this final collated coding framework.

## 5 FINDINGS

Before proceeding to the main qualitative analysis, we present some quantitative analysis of the loneliness question included in the survey to foreground the later analysis. We compare the proportions of those who were lonely at least some of the time by whether they were on or post‐lockdown, on a range of demographic factors identified as relevant to loneliness in the literature above. Results can be seen in Table 2 below.

**TABLE 2:** Lonely at least some of the time (min. 1–2 days per week), by lockdown status – in lockdown (*n* = 493), post‐lockdown (*n* = 1302) and total sample (*n* = 1795)

	In lockdown % (*n*)	Post‐lockdown % (*n*)	Total sample^a^ % (*n*)
	47.9 (236)	41.3 (538)	43.12 (774)
Subsamples			
Gender			
Male	49.0 (47)	39.5 (184)	41.1 (231)
Female	46.5 (181)	42.0 (340)	43.5 (521)
Non‐binary/other	100 (8)	51.9 (14)	62.9 (22)
Physical disability	50.0 (8)	48.9 (22)	49.2 (30)
Carer	52.6 (20)	41.4 (58)	43.8 (78)
Income at the start of COVID			
>$16,000p.a.	51.9% (14)	54.0 (34)	53.3% (90)
<$30,000p.a.	61.0 (25)	49.7 (80)	52.0% (105)
< $56,000p.a.	45.6 (31)	37.5 (78)	39.5 (109)
<$88,000p.a.	50.0 (48)	42.9 (106)	44.9 (154)
<$125,000p.a.	45.5 (46)	40.2 (80)	42.0 (126)
$125,000p.a.+	47.2 (58)	37.4 (117)	40.1 (175)
Social Network			
Reported multiple strong ties pre‐COVID	47.1 (131)	37.1 (260)	40.0 (391)
Lacked multiple strong ties pre‐COVID	48.8 (105)	46.2 (278)	46.9 (383)
Relationship Status			
In a relationship	44.0 (139)	34.7 (274)	37.4 (413)
Not in a relationship	54.8 (97)	51.5 (264)	52.3 (361)

^a^ 1795 participants answered this 1‐item question on loneliness.

The findings support the relevance of the RQ; not only did a greater proportion of those in lockdown report more lonely days than those who had exited it, but there is evidence of heightened residual loneliness – or persistent loneliness gaps – for several groups. Men and those with carer responsibilities fared worse during lockdown, but readjusted quickly post‐lockdown, with reduced likelihood of loneliness. However, those with a physical disability, low income or who lacked multiple strong ties per‐COVID had both higher levels of loneliness in lockdown and persistent loneliness post‐lockdown.

Moving onto the qualitative findings, we found that changes wrought by COVID‐19 were fundamental to the activities, connections and experiences of marginalisation of participants. Three broad themes emerged: 1) “*It's just not the same*” portrays the transition to digital communication on socioemotional well‐being during and post‐lockdown, with clear unequal impacts across social strata; 2) “*Renegotiated relationships and networks*” shows how social networks have become more insular and bonding oriented, with negative repercussions for those lacking social capital; and 3) “*Life course interrupted*” illustrates how education, work and living arrangements shifted online and changed life trajectories, with implications for long‐term disconnection and loneliness for key groups. Concerns raised in the literature over the acceleration of late modern trends in family, work and social connection during and after COVID‐19 were apparent to different degrees in the findings.

### 5.1 “It's just not the same”: the socioemotional impact of COVID‐19 depended on digital skills and preexisting conditions

There was a general sense of rising disconnection during lockdown, linked to closure of usual activities and associated networks, which a capacity for online contact did not ameliorate. Examples of lost activities included “music gigs” (Female, 24), “martial arts” (Male, 28) and “dancing” (Female, 33). As the following accounts show, restrictions affected both intimate and social attachments, appearing to increase social and emotional loneliness:

Devastated. I go for weeks without seeing friends and loved ones face‐to‐face. Online alternatives help a lot, but it's not the same and not enough. (Female, 36, in lockdown)

Being a volunteer for more than 18 years, COVID‐19 closed all of that down since March 2020. The lack of social interaction during this time affected me mentally. (Male, 74, in lockdown)

Participant’s pre‐COVID‐19 digital resources, skills and experiences impacted the ease with which they transitioned to online communication during lockdown. Those experienced with carrying out relationships online described being protected against the potential social losses of the transition:

I have for decades had many online relationships all over the world…This has facilitated my ease at moving to online. (Female, 70, in lockdown)

Others noticed little difference because much of their socialising was already online. The following account reflects this sentiment, representative of a generally younger group of respondents: “Most of my other social groups (e.g., I am in some hobby groups) were online‐only to begin with, so they weren't really affected” (Female, 24, 77 days).

In contrast, participants lacking digital and social skills described the transition negatively. One who had “never been an online person” felt the digital format tarnished their social interactions: “I hate chatting ‘cause I'm a slow typer. I hate Skype in part because I hate seeing myself on screen [and] hate other people seeing me” (Female, 36, in lockdown). Another reported that the “digital format” had exacerbated their longstanding social “discomfort” and they had “tended to avoid most social contact since working from home” (Female, 31, in lockdown), suggesting that for some, not socialising was preferable to poor‐quality digital interactions. The perception that others were coping more easily created additional anxieties, and exacerbated isolation:

I have difficulty connecting with people online, so it has been an isolating experience because I keep hearing how others are always staying connected via these methods. (Female, 28, in lockdown)

Those who experienced such digitally driven disconnection felt it lasted beyond lockdown, interacting with concerns over the risks of groups returning face‐to‐face. Accounts also reflected rising social and collective loneliness (Cacioppo et al., 2015), as implied in a perceived loss in motivation and activities central to one's identity:

Everyone became withdrawn, even after restrictions ended. No one wants to hang out anymore, everyone feels depressed and down. Feels like life and society have permanently changed even after most of the pandemic has ended…You can make plans and act towards them, but they can (and usually do) come undone in moments. (Male, 34, 230 days)

Several respondents reported high anxiety about the ongoing health impacts of the virus, even post‐lockdown, leading them to continue relying on digital communication. This anxiety led them to avoid meeting others and led others to fear meeting them, creating a feedback loop that reinforced their loneliness, and compounded the experience of living alone:

It has been harder to socialise as I do not feel comfortable going out. This has meant online only, except for immediate family.(Male, 44, 215 days)

I have become more insular, avoiding people that fear contact with me. Living alone created a barrier with COVID making contact that I need very difficult.(Male, 75, 228 days)

Experiences of digital interaction depended on preexisting conditions.

Some self‐described introverts reported increased comfort, stability and control after lockdown, and thriving in the emerging, but still curtailed world. For example, one participant noted: “I'm an introvert and suffer social anxiety, so it's helped in that I'm not expected to do as much social stuff” (Male, 56, 229 days). Several participants with disabilities also gave positive accounts; one participant who was deaf, felt “normal on Zoom” because of the benefits of interacting digitally:

I am equal on Zoom, which has been a blessing! I have had more time for in depth conversations too, a good thing. (Female, 65, 86 days)

However, for many with mental or physical disabilities, the isolation seemed a continuation of a “normal” isolated life pre‐COVID‐19:

“When you are a mentally ill person who rarely leaves (or wants to leave) the house nothing much changed. All that shit where people felt isolated and alone and stuff is just absolutely normal for people like us.” (Male, 40, 51 days)

Others with chronic physical disabilities described how time spent in COVID‐19 restrictions had exacerbated their existing isolation and how their “socialising [had] been reduced to practically none” (Female, 21, 70 days). Participants with preexisting medical conditions, or caring for others that had them, were also hesitant to reengage due to higher health risks of infection. One participant (Female, 28, 79 days) noted: “I have a terminally ill parent and that has taken precedent over other socialising, even before COVID‐19 but especially after.”

### 5.2 Relationships, friendship and networks re‐negotiated (from bridging to bonding)

#### 5.2.1 Changing activities changed networks

Isolation prompted participants to cease physical activities and transition them online. Sometimes this fostered new social contact in support of the “stimulation” hypothesis (Nowland et al., 2018) that digital technology can encourage new forms of connection:

We stopped weekly dinners…[but] we are now playing Dungeons and Dragons, as one of our co‐workers’ boyfriend…is a Dungeon Master. It's been really fun, I really missed our game nights [when lockdown ended]. (Female, 32, in lockdown)

The online diploma I have enrolled in has really made me feel connected to a new group with regular zoom meetings/lectures. (Female, 43, in lockdown)

Not all previous activities were easily transferred online, with restrictions hampering access to substitutes among several older participants:

[I enjoy] attending classical concerts and theatre. I have had to switch to online alternatives and definitely miss the experience of dressing up and going out and the atmosphere of being in an auditorium with many other people as well as the buzz of live performance. (Female, 49, 71 days)

The sense of loneliness has never been stronger…[I normally] travel internationally a lot throughout the year to see all my friends…With bars etc closed for most of the year, I was unable to go meet new people. (Male, 39, 215 days)

These reductions in physical activity represent a substantial reduction in bridging social capital (Patulny & Svendsen, 2007; Putnam, 2000) continuing post‐lockdown while restrictions lasted. To the extent that these remain in place, they may represent a long‐term diminution of informal social networks and habits sustaining such activities.

#### 5.2.2 Closeness and consolidation

There was evidence the initial transition to digital interaction changed the shape and content of participants’ social networks. Many noted that lockdown reduced the quantity of their interactions, and hobby and meet‐up groups were discontinued. It was very common for participants to describe a “pruning” of friendship networks in moving to digital interaction:

It has all gone online and means I don't have as many social interactions as I did [previously] by a long way. I rely on a smaller pool of people for regular social interaction. (Female, 33, in lockdown)

Some people I’m much closer to than I ever have been, some people I’ve lost contact with. (Female, 35, in lockdown)

As time passed after lockdown, this process became one of increasing closeness and consolidation:

I have not seen people in my wider circle of friendship since March shutdown. But I have found that the quality of my friendships has been better with the close few. (Female, 37, 76 days)

“I spend more time with close friends. Less time with ‘acquaintances’. More time with reliable colleagues. Less time with ‘time‐wasters’.”(Male, 60, 228 days)

Several respondents described their core social networks as “completely different” post‐lockdown, with several older participants falling back on preexisting relationships with close friends/family, and a “deterioration or intensification” of different relationships based on their “low or high quality.” It suggests a broad “cocooning” movement (Putnam, 2000), indicative of a retreat away from bridging capital in favour of shoring up bonding capital:

Superficial relationships have disappeared, and the circle of close friends and associates has grown a bit bigger. (Female, 51, 79 days)

[I] feel like I'm drawing down on 'friendship capital', social capital that was developed over the years and it is not being topped up. For closer friends, there is a critical mass where even the online interactions strengthen the friendship to a degree that it is then self‐sustaining. (Male, 35, 88 days)

This “pruning” effect sometimes extended to friendships formed from the emotional “common experience” and “shared anxiety” of the pandemic. Others simply became closer with those they spent the most time with, often work colleagues:

I value work friendships even more now because they are some of the only other people, I have spent time with over the last few months other than my partner (Female, 27, in lockdown)

Many participants concentrated their socialising time among family members, and described them as feeling more important during this time:

“playing a lot more board and card games with family. Cooking and eating as a family became important.” (Female, 60, 71 days)

However, concentrated time with family did not always foster improved closeness or importance, especially if these relationships already were poor quality. As one participant noted “I don't really get along with my parents who I live with, having them around all the time was also really tough for me.” (Female, 33, 76 days).

The pruning of social networks also led to negative socioemotional impacts for those who were “pruned.” There was a sense of disappointment and frustration about friendships unravelling during lockdown, particularly among males. These sentiments mirror research showing the relative fragility of male friendships, often oriented around limited work or leisure activities (Patulny, 2012), that are particularly vulnerable to the cessation of face‐to‐face contact:

I have stopped a lot of friendships and relationships because many of them don't believe in COVID…This has made me sad, and at times angry. (Male, 62, 216 days)

I lost lots of friends. Had a big friend group at work, but since that work got closed down all disappeared. Also, childhood friends have become more depressed and no longer do any activities. (Male, 34, 230 days)

Others described the potentially fatal impact online mediums could have for more distant relationships, prompting concern and sometimes a threat to their agency over social connections:

For more distant groups, the social capital is being drawn down from and isn't replenishing. If the pandemic goes on long enough these groups/relationships may break. (Male, 35, 88 days)

[When talking online], I feel like it is far easier for people to ignore and forget about me when we don't see each other in person… I feel very lonely, and like I have no real way to properly fix that.(Female, 26, 76 days)

#### 5.2.3 Neighbourhood disconnection

Participant accounts provided evidence that the shift to digital interaction ruptured neighbourhood‐based networks. Some suggested the “local” aspect of some relationships lost relevance when digitalisation enabled connection to any location:

I am in touch with friends who live overseas more frequently compared to before COVID‐19, as 1km away and 10,000km away doesn't make much difference right now. I try to catch up with my local friends through the Zoom, but it is not the same. (Female, 31, in lockdown)

This dynamic of the “loss of the local/physical” in favour of the “distant/digital” remained in some relationships post‐lockdown:

Socialising in person has fallen away in most cases, however socialising digitally has actually become easier and more frequent as it has been easier to find times where everyone has time for a video call. This also made it easier to keep up with overseas friends and family. (Female, 33, 77 days)

There was further evidence of participants disconnecting from in person groups and the “effort” required to connect online once the local context of these interactions was removed. One participant noted: “It's made me realise the ‘local’ friends who I really hang out with just because they are convenient” (Female, 31, 79 days).

Not everyone had existing networks to draw on during lockdown, particularly those with low prior social capital/support. Prior studies have linked reduced community engagement to loneliness (Kraut 2002), and several participants described such situations, and having few close friends nearby:

I didn't have many friends before…It would be nice to be a part of a social group but that is a bit hard at the moment with restrictions and I also can't find one.(Female, 24, 90 days)

Such findings demonstrate that rather than experiencing a wholesale loss in connections and increased loneliness, many instead consolidated networks, and shifted from broad, locally focussed bridging networks toward more selective, online, bonding networks fulfilling particularised needs. This might assuage emotional and social loneliness through improved family connections, although at a cost of potentially increased social and collective loneliness through losing more distant community connections.

### 5.3 “Life course interrupted”: positive/negative changes in life trajectories

While COVID‐19 may have only suspended some networks and activities, there were concerns about the longer‐term impacts on life goals and opportunities. One respondent observed how the enforced isolation fractured new romances and raised anxiety about securing future partnerships:

Meeting someone feels pretty impossible this year, which I’m a bit anxious about it because I’m so conscious about being 35. (Female, 35, in lockdown)

While this experience might primarily be attributed to lockdown, numerous responses in the months post‐lockdown suggested many experienced more substantial life course “interruptions.” For example, COVID‐19 interrupted the trajectories of people who were about to start important new life phases, such as relocation for education and work or trying to form a new identity:

[COVID‐19] came when I was already depressed and just beginning to try really hard to build a social network…. [it] really delayed my start in building a new social network because the hobby club I joined could not meet…I feel overlooked because I am unusual, with no children or partner. (Male, 62, 55 days)

I had recently moved so I was still in the process of making friends, that kind of had to stop so there is a sense of stalling. I am pretty sociable but I couldn't make enough friends to satisfy me and I don't know when that will change so that’s distressing. (Female, 33, 76 days)

Due to COVID I haven’t been able to make friends at university, as I started post‐grad this year and from the 2nd week of uni everything was moved online. Being in a new state this has been particularly hard for me. (Female, 23, 90 days)

Longer‐term implications of such changes are unclear. They might represent a long‐term interruption of late modern work and mobility patterns (Urry, 2000), linked to social alienation and loneliness (Bauman, 2005; Sennett, 1998). However, while participants regularly reported working from home, there was little reporting of increased take‐up of gig work or other online opportunities optimistically promoted as positive side effects of the pandemic (Nagel, 2020; Spurk & Straub, 2021).

Furthermore, these disruptions were accompanied by a ceasing of everyday leisure activities and a “normal” routine to fall back on. Some who managed to return to regular church and playgroup activities “social distancing‐style” described them as “very frustrating to have to work within the restrictions” (Female, 39, 75 days). Others noted a general fatigue and apathy with trying to revive community life and activities, and “unlearning” the habits of in person sociability in favour of digital interaction:

Now that things are opening up I am finding that I don't want to come back out. I trust people a lot less… I actually don't want to make the effort any more to really connect(Male, 60, 230 days)

It was weird how everyone still stayed at home even after restrictions lifted. Social activities are all but dead. (Male, 34, 230 days)

Now restrictions have lifted but face‐to‐face is preferred [and] it feels tiring. It's changed for us all. (Female, 51, 72 days)

Another common disruption was the impact of moving back home, for (mostly) younger persons lacking resources to survive independently during COVID‐19. While media attention focused on youth returning to family homes to draw on family support and save on housing costs (Pinsker, 2020), less attention is paid to the life disruptions these people experience. The participant (noted above) felt “really lucky to be at my parents place when coronavirus hit because it’s been so good to have the company” (Female, 35, in lockdown). However, the participant (conscious of lacking a relationship at 35) reported a loss of connection to friends, and constraints in meeting new partners, particularly with health concerns over elderly parents:

I saw friends for one outside coffee in May, but apart from that I haven’t seen any friends in‐person since March because I’m back living with my parents… they’re both 69 years old and they also see my grandma who’s 95 so I’ve been trying to be as cautious as possible.

These findings suggest that while returning home might improve family bonds that can reduce emotional loneliness (Das, 2021), it may also increase social and emotional loneliness through disconnection from friends and potential partners, representing another contributor to a widening age gap in loneliness.

Finally, participants described the disruption that COVID‐driven unemployment had on their social world and identity. One participant described the loss of a music group that was: “very important for my self‐worth … because I was unemployed, so I needed this hobby to feel worthwhile.” (Female, 33, 76 days). Unemployment and subsequent loss of networks also had repercussions post‐lockdown:

“[I] lost two jobs, and all the social relationships involved in those. Method of socialisation is returning to 'normal', less eating out of home or going to bars/restaurants, given that only a handful of my social relationships have regular employment anymore.” (Male, 23, 230 days)

Reports like these reflect pre‐COVID research linking social exclusion and compromised participation in normal social activities to financial downturns (Patulny & Wong,  2013), as well as to the marginalisation and loneliness faced by long‐term unemployed or low‐income people (De Jong Gierveld et al., 2016; Peterie et al., 2019). Other studies reveal COVID’s disproportionate impact on workers unable to shift to digitised labour, e.g. young hospitality workers (Cook et al., 2021). Our findings suggest COVID‐19 has widened the loneliness gap between those with employment choices (including online vs. offline work), and those precariat/unemployed workers who lack them.

## 6 DISCUSSION

The results show that COVID‐19 had substantial and uneven impacts on the lives of Australians, lasting post‐lockdown. Participants reported increased disconnection and loneliness (social, emotional and collective) associated both with COVID‐19 lockdown and its aftermath, resulting from longer‐term changes in activities and interactions, and reduced freedoms typically associated with late modern lifestyles. There was a sense that interaction was “just not the same” with a perceived “drift” in connection quality and lost physical connection.

COVID‐19 exacerbated existing forms of loneliness‐based marginalisation and created new ones, in ways that continued post‐lockdown. It created additional difficulties for those with physical disabilities, some of whom avoided contact because of higher COVID‐19 health risks and anxiety; this reinforces qualitative findings from other studies linking preexisting health issues to increased COVID‐induced anxiety around social contact (Ratcliffe et al., 2022). For others, it exposed the “normality” of an absence of contact, and the ease with which they “could be ignored.” It revealed new inequalities in loneliness between those with physical and psychological disabilities and their carers, and those with no such pre‐conditions or responsibilities.

In terms of activities, a sense of disconnection and loss remained well past lockdown while social restrictions remained (e.g. gathering for dance/performance groups). Some reported positive experiences of trying new activities and meeting new people online, akin to the Nowland et al. (2018) concept of “stimulation.” However, others reported ongoing feelings of social stagnation, including a general disruption of in person voluntary activities (and declining bridging social capital), a lost “buzz” associated with public socialising and increasing loneliness from being unable to travel and meet new people. Those with poor preexisting levels of social capital or recent ruptures in support (e.g. divorce) felt the absence of activities and connections most keenly. These loneliness inequalities support prior findings about the heightened difficulties created by COVID for single and separated people (Zaninotto et al., 2022) and extend these emerging inequities to the social capital poor.

COVID‐19 lockdown also impacted social networks and capital directly, particularly through the common theme of pruned and consolidated networks. The idea of “pruning” reflects findings from other COVID studies around “narrowed” social spheres (Ratcliffe et al., 2022) or “funnelling” contacts through prioritising and strengthening care, support and communication with some contacts over others (Vrain et al., 2020). However, the “pruning” differs from this earlier phenomenon in that it appears more deliberate – involving decisions to not just focus on some contacts, but to actively end others perceived to be negative – and involves longer‐term consequences for both the pruners and the pruned. Network shrinkage badly impacted those who were “pruned,” most of whom were male respondents.

Digital communication assisted this consolidation, by restricting group size and orienting communication to talking rather than activities, indicative of a “cocooning” effect (Putnam, 2000), and a movement away from bridging and toward bonding social capital. Some perceived this change as positive, shepherding improved quality of interactions with a smaller group, with closeness amplified through shared pandemic intimacy. However, others reported digital interaction to be insufficient and “not enough,” in keeping with findings from other studies (McKenna‐Plumley et al., 2021).

Those lacking experience in digital interaction felt impacts of lockdown more severely, who rejected or became more anxious about digital communication. Younger, digitally literate respondents typically avoided this problem, although some reported near‐complete loss of physical networks, a concerning “displacement” rather than “stimulation” (Nowland et al., 2018) of networks. This, both the digital divide (for older persons) and internet use (for younger persons) were important contributors to the loneliness gap.

While our study reinforces prior findings that while COVID both strengthened home‐based family relationships for some and thwarted new romantic partnerships for others (Ratcliffe et al., 2022), it goes further in identifying how many younger participants’ longer‐term “life trajectories” were disrupted by lockdown, including education and work‐related relocation plans. This might represent an interruption of late modern work and mobility patterns, with a possible arresting of their socially fragmenting effects (Urry, 2000). However, while younger participants who had to move home might have temporarily alleviated loneliness through improved family ties (Das, 2021), they typically reported that such arrangements also constrained friendships, romance and longer‐term travel or occupational plans. We also found a decline in longer‐term habits of sociability, which extends UK findings of increased anxiety among adolescents returning to school or adjusting to “real life” after lockdown (McKinlay 2022) to adults of all ages.

Finally, the quantitative descriptive analysis of loneliness reinforced these qualitative findings, identifying persistent loneliness gaps after lockdown for key groups, including those with a physical disability, low income or low social capital.

## 7 CONCLUSION

COVID‐19 and its aftermath appear to have accelerated late modern physical disconnection, isolation and loneliness (Franklin et al., 2019) in several ways. COVID has opened up new “loneliness gaps,” requiring policy solutions and new research to fully address.

The lasting effects of disbanded social groups, activities and pruning of networks across the board call for helpful interventions. These include community initiatives that reverse pruning and increase bridging capital – such as community grant schemes to build or maintain in‐person community and civic groups, like “men’s sheds,” known to reduce loneliness (Haslam et al., 2016). While these initiatives may not guarantee the quality contact that reduces in social loneliness, those that emphasise repeated, familiar and meaningful interactions are more likely to succeed (Bessaha et al., 2020) and build bonding as well as bridging social capital.

This study shows lockdown directly isolated key groups, including singles, those with social anxiety, physical and mental disabilities (and their carers) or lacking prior social capital. Such groups might require more specialised interventions. For example, interventions targeted at building group connections (e.g. community groups) might suit singles better than individual‐focused interventions (e.g. tele‐services and friend‐lines) (Eccles & Qualter, 2021; Fakoya et al., 2020), while interventions focussed on cognitive techniques might better suit those with anxiety (Masi et al., 2011). Research should explore long‐term effects of the pandemic on these groups.

The study points to the potential problem of a more long‐term shift from physical to digital interaction opening loneliness gaps based on age. The ongoing digital divide among older people with reduced digital literacy raises the need for more elder IT training programmes (Fakoya et al., 2020). For the young, the “stimulation” effect of the digital uptake was clearly outweighed by the “displacement” effect (Nowland et al., 2018) of physical isolation during and after COVID‐19. Younger people were more likely to experience altered life courses along with changing work patterns and priorities. Programmes connecting youth (Eccles & Qualter, 2021), or connecting people to meaningful study or work, (e.g. funded scholarships), or recover lost ties to the workforce, and related social capital, will be timely going forward.

It is unclear how many of these changes will last long term, although the results here suggest that the effects will be unevenly experienced. Those lacking physical health, social capital and digital interactive skills are already more marginalised and at greater risk of loneliness in the post‐COVID‐19 world. While this may not grow into entrenched cultures of loneliness, the pre‐conditions for an expanded “loneliness gap” seem apparent.

The studies’ main limitations suggest directions for future research. The study used a convenience sample rather than representative population sample and inferred different forms of loneliness rather than measure them with validated instruments. It also primarily recruited people through digital means, thereby potentially omitting those who lacked digital connectivity (although the recruitment of extra participants for face‐to‐face interviews did mitigate this to some extent).

We would urge future research employ such data to directly measure changes in the different forms of loneliness herein as we continue to learn to live with COVID. In particular, there should be a focus on those groups most susceptible to the COVID‐induced loneliness gap: singles, those with disabilities, carers, those lacking digital and social capital, older and younger persons, the precariat and unemployed.

## AUTHOR CONTRIBUTIONS

**Roger Patulny:** Conceptualization; data curation; formal analysis; investigation; methodology; project administration; writing – original draft; writing – review and editing. **Marlee Bower:** Conceptualization; data curation; formal analysis; funding acquisition; investigation; methodology; resources; writing – original draft; writing – review and editing.

## Endnotes

^1^
One exception was a major outbreak and second lockdown in the state of Victoria; data from this experience are included in this analysis.

^2^
Face‐to‐face interviews were conducted through services for people experiencing economic exclusion or housing issues to engage people difficult to recruit online, but only a fraction of responses from this group were included after search filters were applied (*n* < 20).
